# Longitudinal tracking of hemocyte populations in vivo indicates lineage relationships and supports neural progenitor identity in adult neurogenesis

**DOI:** 10.1186/s13064-024-00185-3

**Published:** 2024-06-20

**Authors:** Alex J. Edwards, Barbara S. Beltz

**Affiliations:** 1 Neuroscience Department, Wellesley College, Wellesley, MA 02481 USA; 2 The Ken & Ruth Davee Department of Neurology, Northwestern University Feinberg School of Medicine, Chicago, IL 60611 USA

**Keywords:** *Procambarus clarkii*, Crayfish, Hematopoietic tissue, Immune system, Hemocyte lineage, Neurogenic niche

## Abstract

Adult neurogenesis, which takes place in both vertebrate and invertebrate species, is the process by which new neurons are born and integrated into existing functional neural circuits, long after embryonic development. Most studies in mammals suggest that self-renewing stem cells are the source of the new neurons, although the extent of self-renewal is a matter of debate. In contrast, research in the crayfish *Procambarus clarkii* has demonstrated that the neural progenitors producing adult-born neurons are capable of both self-renewing and consuming (non-self-renewing) divisions. However, self-renewing divisions are relatively rare, and therefore the production of adult-born neurons depends heavily on progenitors that are not replenishing themselves. Because the small pool of neural progenitors in the neurogenic niche is never exhausted throughout the long lives of these animals, we hypothesized that there must also be an extrinsic source of these cells. It was subsequently demonstrated that the neural progenitors originate in hemocytes (blood cells) produced by the immune system that travel in the circulation before ultimately integrating into niches where the neural lineage begins. The current study examines the developmental lineage of the three hemocyte types — hyaline (HC), semigranular (SGC) and granular (GC) cells — with the goal of understanding the origins of the progenitor cells that produce adult-born neurons. Longstanding qualitative metrics for hemocyte classification were validated quantitatively. Then, in a longitudinal study, proliferation markers were used to label the hemocytes in vivo, followed by sampling the circulating hemocyte population over the course of two months. Hemolymph samples were taken at intervals to track the frequencies of the different hemocyte types. These data reveal sequential peaks in the relative frequencies of HCs, SGCs and GCs, which were identified using qualitative and quantitative measures. These findings suggest that the three hemocyte types comprise a single cellular lineage that occurs in the circulation, with each type as a sequential progressive stage in hemocyte maturation beginning with HCs and ending with GCs. When combined with previously published data, this timeline provides additional evidence that HCs serve as the primary neural progenitor during adult neurogenesis in *P. clarkii*.

## Background

Adult neurogenesis, which occurs in both vertebrate and invertebrate species, is a process by which new neurons are born and integrated into existing neural circuits after an organism has completed embryonic and early post-embryonic development. Self-renewing stem cells and other pluripotent cells, such as pericytes, have long been the presumed neural progenitors underlying adult neurogenesis in mammals [1, 11, 34]. However, a number of studies in mice have demonstrated that hippocampal and olfactory bulb neural stem cells have a limited life-span in vivo [8, 12, 13], and not the long-term self-renewal that has been proposed. Such findings suggest that these organisms would require a large reservoir or ongoing source of neural progenitors in order to sustain adult neurogenesis during the long lives of many mammalian species.

**Fig. 1:**
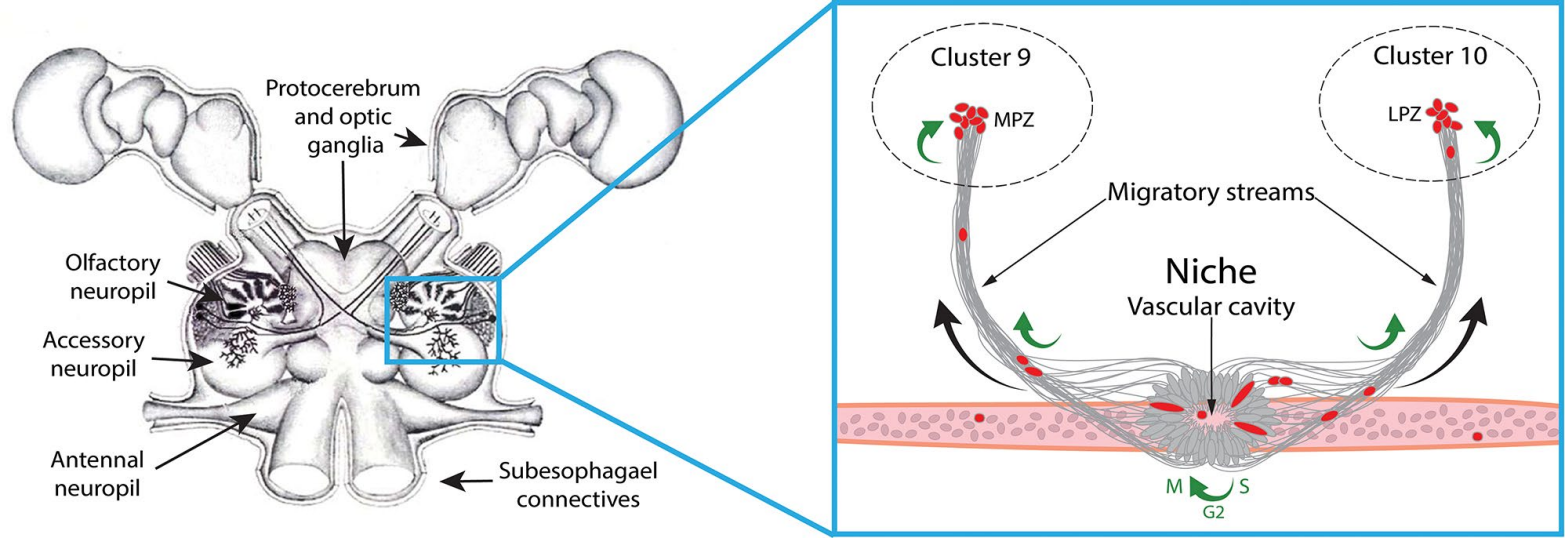
**Schematic representation of neurogenic niche.** **Left:** Drawing of the *P. clarkii* brain (supraesophageal ganglion), showing the eyes and protocerebrum, deutocerebral (olfactory and accessory) neuropils and neuron clusters, tritocerebral antennal neuropils and connectives leading to the subesophageal ganglion. The blue box demarcates the area of the brain illustrated in the right panel. **Right: **Diagram showing the neurogenic niche, streams and blood vessel on which these are situated. Red oval cells are located in the niche, where they divide. Descendants of consuming divisions then move into the migratory streams, which are composed of the long projections of bipolar niche cells. These cells divide while in the streams, along which they migrate to the medial and lateral proliferation zones (MPZ, LPZ) in Clusters 9 and 10, respectively. Curved green arrows represent locations of some observed mitotic divisions; curved black arrows represent the direction of movement of neural progenitors in the streams. S, G2, M: stages of mitosis that occur in the neurogenic niche. Left panel adapted from Benton et al., 2013; original drawing by D. C. Sandeman

In many crustacean species, adult neurogenesis occurs within bilateral neurogenic niches located on the ventral surface of the brain [29]. In crayfish, each niche is composed of bipolar cells whose cell bodies and short processes form the niche itself, and whose long processes make up migratory streams that lead to proliferation zones in the brain (Fig. 1) [35]. Proliferation markers, such as 5-bromo-2’-deoxyuridine (BrdU) and 5-ethynyl-2’-deoxyuridine (EdU), have been used to track neural progenitor behavior within the niche, demonstrating cell divisions in the niche and unidirectional movement from the niche to the proliferation zones via the migratory streams (Fig. 1) [4]. The neural progenitors in the niche are capable of both self-renewing and consuming (non-self-renewing) neurogenic divisions. However, the self-renewing divisions are relatively rare (observed in ~ 5% of preparations [7]), and therefore the production of adult-born neurons must depend heavily on progenitor cell divisions that are not self-renewing. The dominance of consuming divisions is supported by earlier timed studies following injections of proliferation markers showing that the niche cells generally do not retain BrdU, as would be expected if they were predominantly self-renewing. Instead, following cell division in the niche, the BrdU-labeled cells migrate out of the niche to proliferation zones within neuron clusters where progenitors divide again and mature [4, 6]. The niche contains only a few hundred progenitor cells even in mature crayfish [39], with roughly one cell dividing each day [6]. We therefore hypothesized that the system would be rapidly depleted of progenitors — even with a small contribution from intrinsic self-renewing divisions — unless there is an external source of neural progenitors capable of replenishing the niche [2, 3, 7, 39]. This idea is also supported by the finding that in vivo incubation of crayfish with physiological concentrations of serotonin in pond water, which results in heightened levels of neurogenesis, results in a sizable increase in the number of niche cells without any increase in niche cell divisions [6]. The influx of extrinsic cells to the niche is therefore a significant event that can be readily demonstrated.

In the search for a tissue source for neural progenitors in crayfish, several cell types were tested [6]. Both cell culture and adoptive transfer experiments revealed that hemocytes are attracted to the niche and can give rise to neural progenitors [4–6]. This conclusion is consistent with the specific location of the neurogenic niches: directly atop a vascular cavity that is in communication with the circulation [9, 35, 39]. Therefore, hemocytes appear to readily access the neurogenic niche while circulating in the hemolymph, although the mechanisms involved are not known. Mechanisms regulating the proportion of self-renewing vs. consuming divisions also are unknown.

### Crayfish hemocytes

Each of the three hemocyte types in crayfish — hyaline cells (HCs), semigranular cells (SGCs) and granular cells (GCs) — can be characterized based on a variety of quantitative and qualitative morphological characteristics, such as whole cell area, ratio of nuclear area to whole cell area (nucleocytoplasmic [NC] ratio), presence of granules and presence of pseudopodia — as well as on their functional properties [15, 20, 31]. In *P. clarkii*, HCs are characterized by their relatively small cell size and spherical shape, high NC ratio (> 0.50) and lack of cytoplasmic granules. SGCs represent the middle ground of the three hemocyte types: median cell size, median NC ratio (0.30–0.45) and a modest number of granules. These cells tend to have an elliptical or spindle shape, with minimal amounts of pseudopodia. GCs are on the other end of the spectrum, displaying a very large cell size, low NC ratio (0.20–0.35) and an abundance of granules [16, 23]. These cells also can be morphologically identified by their irregular pseudopodia.

Since hemocytes are generated by the immune system (comprised of the hematopoietic tissue [HPT] and the anterior proliferation center [APC]), they are intimately engaged in the immune response in addition to any role in adult neurogenesis. The HPT is known for its production of hemocytes, while the role of the APC is not understood [26]; however, like the HPT, the APC rapidly releases cells into the hemolymph [5]. The identity of those cells is not known, although it has been proposed that some may act like stem cells [9, 26, 33].

The different hemocyte types are also associated with distinct roles in the immune response. HCs have been associated with phagocytosis [16, 23]. The largest number of known immune functions have been attributed to SGCs, which facilitate melanization, encapsulation and coagulation [16, 23]. Lastly, GCs have been identified as being responsible for cytotoxicity and melanization [16, 23].

It is important to note that the cell type in *P. clarkii* that closely fits the published criteria for HCs [15, 20, 23] also undergoes frequent cell divisions. This feature may generate questions about their identity, since these would not represent fully differentiated hemocytes. As Söderhäll (2016) [31] commented, “the nature of HCs has been debated; these cells may be immature or prematurely released prohemocytes of the SGC or GC lineage…”. Indeed, Roulston and Smith (2011) [27] defined a fourth cell type (in addition to HCs, SGCs and GCs) in the spider crab *Hyas araneus* that undergoes cell divisions, and could be separated and distinguished from all other hemocytes using Percoll gradients and flow cytometry. They named these “prohemocytes”, however, they also express doubt about the cells’ identity, commenting that their status as immature hemocytes “needs to be clarified” [27]. In the current study, the mitotically-active cells in *P. clarkii* fit the previously published qualitative and quantitative criteria for HCs — and we are therefore identifying these as HCs, with the understanding that they may play multiple roles as they mature, leave the cell cycle and undertake the roles in immunity that have been reported previously.

### Hemocyte lineage models

There is also debate about the lineage relationships among the three types of hemocytes. Previous studies have suggested that these mature along two cellular lineages, in which hematopoietic cells differentiate towards either an SGC or GC fate before release from the HPT and then achieve that fate once in the circulation [23]. This type of branching lineage is present in insects and other crustaceans — *Drosophila melanogaster*, *Anopheles gambiae*, *Manduca sexta* and *Penaeus monodon* [14, 19, 21, 25, 36]. However, recent in vitro studies have challenged the conservation of these lineages in crayfish, instead suggesting that hemocytes participate in a single developmental lineage. In vitro work by Li et al. (2021) [22] in the crayfish *Cherax quadricarinatus* indicates that hemocytes are released from the HPT as SGCs, reaching full maturity as GCs after one to three months, at which point they can survive for up to two more months. As a result of these conflicting studies and conclusions in crayfish, the lineage relationships among the three hemocyte types is controversial.

### The present study

The studies described here have three aims. First, two quantitative metrics for categorizing hemocytes (cell area and NC ratio) were validated as reliable predictors of hemocyte type that are aligned with qualitative hemocyte features. Second, the proliferation marker EdU was injected into live crayfish and hemolymph samples were taken at various time points over the next two months. Only cells in S phase at the time of injection would be labeled with the proliferation marker following sample processing. This in vivo longitudinal study, therefore, allows the tracking and observation of the EdU-labeled hemocyte population over time, enabling quantitative observations of large numbers of EdU-labeled hemocytes. The resulting data have allowed us to develop a hemocyte lineage model for *P. clarkii*. Third, by determining variations in the frequencies of the hemocyte types over time and comparing this timeline with previously published data related to adult neurogenesis, these experiments inform our understanding of the hemocytes that act as neural progenitors, confirming previous data indicating that HCs are the most likely source of adult-born neurons in this crayfish [4, 5].

## Methods and materials

### Animals

Male (*n* = 3) and female (*n* = 3) adult crayfish (*P. clarkii*; 25–35 mm carapace length [CL]), were used in these experiments, although not all crayfish contributed to the longitudinal experiment (*n* = 4). An additional male crayfish was used as a control. All animals were originally sourced from Carolina Biological Supply Co. (Burlington, NC), and maintained and bred over several months in the Wellesley College Animal Care Facility. Crayfish were housed in tanks containing recirculating aerated artificial pond water, a shallow layer of pea stone, plastic “plants” and PVC tubes and ceramic mugs for shelter and habitat enrichment. They were kept at room temperature (18–20ºC) on a 12:12 h light:dark cycle (7 AM lights on; 7 PM lights off), and were fed 3 times/week with shrimp pellets and fresh carrots.

### Total hemocyte counts

Prior to the experimental use of each animal, total hemocyte counts (THCs) were determined to ensure that each animal included in the study had a normal baseline hemocyte density relative to its size, as determined by Kelley (2018) [18]. Hemolymph was drawn from crayfish using a 25 gauge 5/8’’ ice-cold needle inserted into the dorsal sinus; approximately 30 µl of hemolymph was allowed to collect in the needle hub before the needle was removed. Then, 10 µl of hemolymph was immediately transferred to an Eppendorf tube on ice containing 20 µl anticoagulant (AC) buffer (0.14 M NaCl, 10 mM EDTA, 30 mM trisodium citrate, 26 mM citric acid, 0.1 M glucose; pH 4.6) to prevent clotting. This solution was mixed 1:1 (vol:vol) with trypan blue dye (ThermoFisher Scientific, #15250061) to label any dead cells, before being transferred to a disposable 4-chip hemocytometer (Bulldog Bio, Inc., Portsmouth, NH) for quantification (https://www.bulldog-bio.com/wp-content/uploads/2018/05/4_Chip_User_Manual.pdf). Final THC values represent the number of cells present in 1 ml of circulating hemolymph. All animals used in the current experiments were confirmed at the beginning of the study to have THCs within expected levels based on CL, since THC is positively correlated to carapace length and body weight [18].

### In vivo labeling of hemocytes with proliferation marker

Animals were injected in the abdominal flexor muscle with the S-phase marker EdU (Invitrogen, #C10639; 10 µl/g body weight of 0.1 mg EdU/ml crayfish saline (205 mM NaCl, 5.4 mM KCl, 34.4 mM CaCl_2_, 1.2 mM MgCl_2_, 2.4 mM NaHCO_3_) at 8 AM, and then returned to holding tanks in the Animal Care Facility. Hemolymph was drawn from each crayfish at intervals post-injection (6 h and 3, 10, 17, 31, 45, 56 and 63 days). An additional uninjected male crayfish was used as a control to determine if the EdU injection altered proportions of hemocyte types in matching timed hemolymph samples.

For each time point, hemolymph was collected from the crayfish using a 25 gauge 5/8’’ ice-cold needle coated with AC buffer. All hemolymph draws occurred at 8 AM, with the exception of the 6 h time point. Hemolymph was drawn from the dorsal sinus directly into a 1 ml syringe containing AC buffer, to a final ratio of 1 part hemolymph:1 part AC buffer. The contents of the syringe were transferred to a sterile Eppendorf tube and gently mixed. Immediately thereafter, 100 µl of the hemolymph sample was transferred onto a slide precoated with 0.01% poly-L-lysine (Sigma-Aldrich, #P4707) to promote cell adhesion. Three slides were created per time point per animal, including the uninjected control. Cells were allowed to adhere to the slide for 30 min before rinsing with crayfish saline. Hemocytes were fixed for 10 min with 4% paraformaldehyde (Boston BioProducts, Inc; #BM-155) in phosphate buffered saline (PBS; 20 mM NaH_2_PO_4_, 80 mM Na_2_HPO_4_; pH 7.4). Following fixation, the slides were immediately incubated for 30 min in the Click-iT® Plus reaction cocktail containing the Alexa 594 fluorophore (Invitrogen, #C10639). Cells were rinsed with PBS containing 0.3% Triton X-100 (Sigma-Aldrich, #X100) (PBTx) and incubated overnight in mouse anti-tyrosinated tubulin (1:1000 in PBTx; Sigma, #T9028).

The next day, slides were rinsed with PBTx and incubated overnight in goat anti-mouse conjugated to Alexa 647 (1:100 in PBTx; Jackson Immunoresearch Labs, West Grove, PA, #115-605-166). Slides were washed with PBTx, incubated in Hoechst 33342 for 30 min (1:2000 in PBS; ThermoFisher Scientific, #H3570) and rinsed with PBS. A glass coverslip was mounted on each slide with FluoroGel (Electron Microscopy Sciences, Hatfield, PA) and slides were stored at 4ºC.

### Image analysis

Cells and labeling were visualized and images were collected using a Leica TCS SP5 confocal microscope equipped with 488 nm argon and 543 and 633 nm helium-neon lasers. To assess the proportion of EdU-labeled cells (overall proliferation frequency) for each animal, at least 300 cells were surveyed from each of the three slides prepared per animal. For each slide, parallel rows of cells were imaged, moving horizontally on the slide; all Hoechst-labeled cells in a single row were counted until the total reached or surpassed 100 cells. Then, the region of interest was returned to the start of the first row and adjusted downward two full frames, such that the parallel rows were spaced 150 μm apart to avoid double-counting any cells. This process was repeated until three rows of at least 100 cells were imaged per slide, with rows separated by at least 150 μm. To calculate the hemocyte proliferation frequency for each animal at each time point, the total number of EdU-labeled cells was divided by the total number of Hoechst-labeled cells (EdU + and EdU-). All images were taken using the Leica LAS X software at 63X magnification.

Issues with cells not sticking to the slides during the settling period or with incomplete antibody labeling rendered some slides unusable, and so some animals had only two slides for analysis for a given time point. This variability was factored into subsequent data analysis by normalizing all data as an average of the percentage of EdU-labeled hemocytes relative to all cells (both labeled and unlabeled) across all available slides per time point.

After confocal imaging, individual EdU-labeled cells were assigned a cell type based on qualitative characteristics, such as relative cell size, cell shape and presence of granules. Following this assessment, ImageJ [30] was used to trace nuclear areas and whole cell areas, in order to calculate the NC ratios. Every EdU-labeled cell counted (*n* = 2843) was analyzed for all animals. EdU-labeled cells were excluded from the analysis only if their outline, visualized using the Nomarski filter and tubulin labeling, was not definable due to close proximity to or overlap with surrounding cells.

### Statistical analysis

All statistical analyses were performed in R. First, the Shapiro-Wilk and Levene’s tests were performed on all data to test for normal distributions and equality of variances, respectively. This revealed non-normal distributions across the data and heterogeneity of variances. Due to these results, the Kruskal-Wallis test followed by Dunn’s test for multiple comparisons with Bonferroni’s correction were used for comparing groups. Confidence intervals were calculated using non-parametric bootstrap sampling. Correlations were determined using Spearman’s rank correlation test. Associations between cell types in the longitudinal study were determined using the Chi-square test and subsequent Cramer’s V test. Significance refers to *p* < 0.05. Data are presented as mean ± S.E.M in all figures. GraphPad Prism 10 was used for graph generation.

## Results

### Quantitative hemocyte characteristics strongly correlate with hemocyte type defined qualitatively

**Fig. 2:**
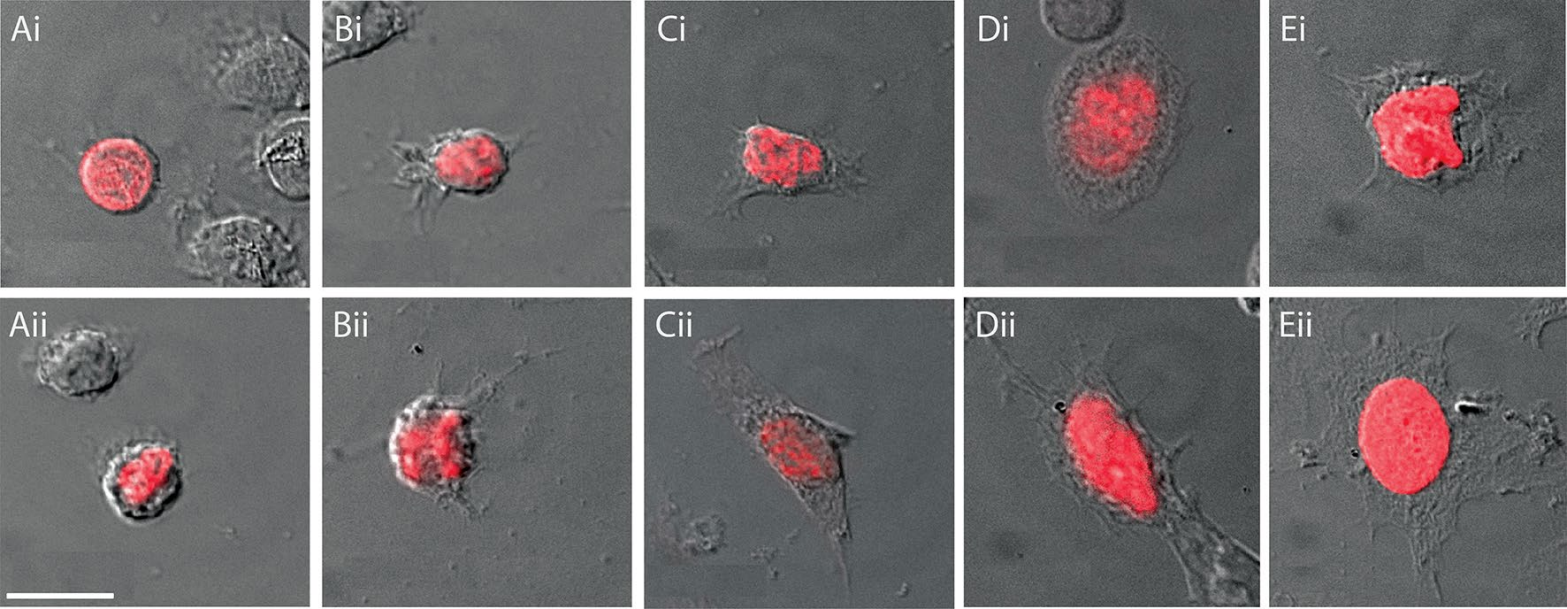
**Each hemocyte type has distinct morphological features.** Examples of the three major hemocyte types (HC, SGC, GC) and transitional forms (HC/SGC, SGC/GC). Images are from 0.5 μm thick sections taken on Leica LAS X software. Cells were visualized using the Nomarski confocal filter and the 633 nm diode laser. EdU (red) labels the nucleus in each hemocyte. Scale bar in (**Aii)** represents 10 μm in all images. **(Ai, Aii)** HCs. **(Bi, Bii)** HC/SGCs. **(Ci, Cii)** SGCs. **(Di, Dii)** SGC/GCs. **(Ei, Eii)** GCs

The first aim of this analysis was to validate the longstanding quantitative metrics of the hemocyte classification scheme — NC ratios and whole cell area — as initially described for decapod species by Hose et al. (1990) [15] and confirmed and extended in *P. clarkii* by Lanz et al. (1993) [20]. To accomplish this, *P. clarkii* crayfish were injected with EdU and hemolymph samples were taken such that three slides were processed per animal as described above (Methods), resulting in over 2800 total EdU-labeled cells and over 33,500 non-EdU-labeled cells surveyed with confocal microscopy and Nomarski optics. Each EdU-labeled cell was first categorized as a specific hemocyte type based on qualitative features such as relative cell size (smallest, intermediate, or largest of the three hemocyte types), morphology and presence or absence of granules (Fig. 2). As this data collection was conducted, it quickly became apparent that not all hemocytes adhere to the discrete categories of HC, SGC and GC. To accommodate this situation, intermediate categories were created and utilized for the remainder of the analysis, noted as HC/SGC and SGC/GC to reflect the cell types they were bounded by and whose properties they share (Fig. 2Bi, ii, Di, ii).

**Fig. 3:**
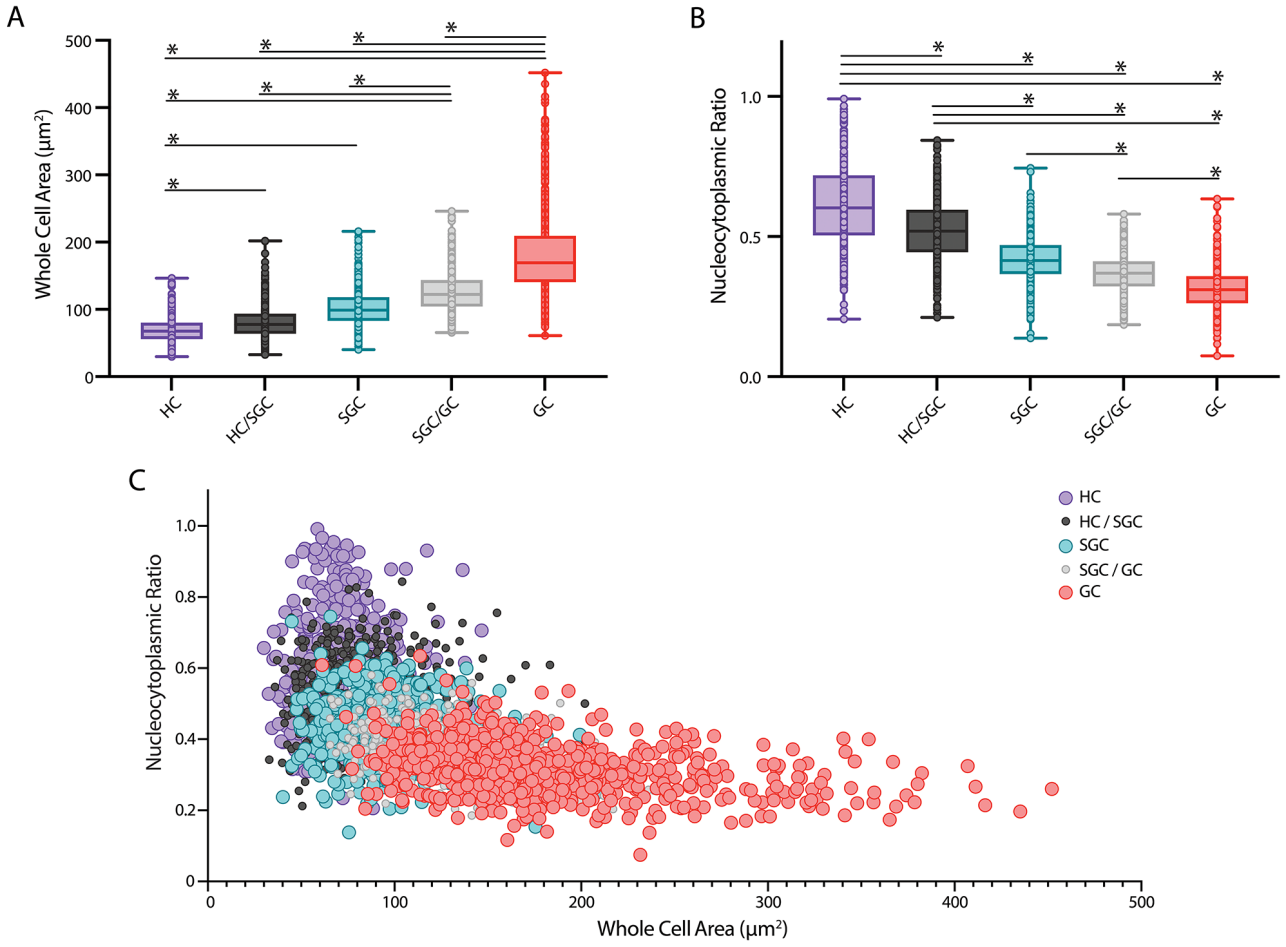
**Different hemocyte types have distinct quantitative and qualitative morphological features.** Hemocytes (*n* = 2843) were categorized by cell type based on aforementioned qualitative and quantitative morphological characteristics. Statistical significance determined by Kruskal-Wallis test and Dunn’s multiple comparisons test with Bonferroni’s correction. **(A-B)** In each boxplot, the central line within the colored box represents the median. Sample sizes for the various hemocyte types are as follows: n_HC_=367, n_HC/SGC_=537, n_SGC_=818, n_SGC/GC_=430, n_GC_=691. Asterisk indicates *p* < 0.05. **(A)** Whole cell area plotted against cell type. **(B)** NC ratio plotted against cell type. **(C)** A strong inverse correlation between NC ratio and whole cell area across hemocyte types is shown (ρ = -0.6367, *p* < 0.0001). Each color-coded point (see key at right) represents one cell, with transitional states represented by smaller black (HC/SGC) and gray (SGC/GC) circles

HCs in *P. clarkii* were the smallest hemocyte type, with an average cell area of 69.93 µm^2^, 95% CI [68.00, 71.89] (Fig. 3A). These cells consistently contained little visible cytoplasm — averaging an NC ratio of 0.619, 95% CI [0.602, 0.635], the highest NC ratio of all the cell types (Fig. 3B). SGCs yielded median values for each of these quantitative metrics: average cell area of 101.97 µm^2^, 95% CI [99.90, 104.00] (Fig. 3A) and average NC ratio of 0.420, 95% CI [0.414, 0.425] (Fig. 3B). GCs on average had the largest cell area (182.45 µm^2^, 95% CI [178.12, 187.05]; Fig. 3A) and the smallest NC ratio (0.314, 95% CI [0.309, 0.321]; Fig. 3B). This group of cells varied most widely in cell morphology, but regularly displayed abundant amounts of both granules and pseudopodia. Further, the measurements of the intermediate hemocyte categories (HC/SGC, SGC/GC) fell squarely in between the respective hemocyte types whose features they shared (Fig. 3A and B). The Kruskal-Wallis test followed by Dunn’s multiple comparisons test with Bonferroni’s correction showed that statistically significant differences existed between each of the five cell categories for both NC ratio (*p* < 0.0001) and whole cell area (*p* < 0.0001). Moreover, there existed a significant negative Spearman’s correlation between NC ratio and whole cell area (ρ = -0.6367, *p* < 0.0001) (Fig. 3C).

Further quantitative analyses demonstrated morphological relationships among the five cell types. Clusters by cell type emerged when the whole cell area and NC ratio of individual cells were plotted (Fig. 3C). HC, SGC and GC clusters fell in relatively distinct regions of the graph and HC/SGC and SGC/GC transitional states clustered in between these (Fig. 3C). The extensive overlap between these cell types suggests a close developmental relationship (Fig. 3C), further supported by the sequential and gradual changes in NC ratio and whole cell area across the five hemocyte types (Fig. 3A, B).These data confirmed that NC ratio and whole cell area were indeed reliable markers for classification of discrete hemocyte types (HC, SGC and GC) as well as HC/SGC and SGC/GC intermediate states. Sexual dimorphisms were not observed; specifically, no significant differences were found between male and female crayfish for NC ratios and whole cell areas per hemocyte type. Further, the relative frequencies of the different circulating hemocyte types were confirmed to be unaffected by the EdU injection when compared to hemolymph from the control animal.

### Mitosis occurs in hemocytes in the circulation

Mitotic stages were observed in circulating hemocytes. These were, however, rare events that comprised fewer than 1% of all hemocytes surveyed. This may suggest a very rapid mitotic cycle, such that mitotic stages were not frequently observed. NC ratio and whole cell area were unreliable metrics in these instances due to the doubling of the nuclear area and subsequent expansion of the total cellular area. However, morphological characteristics — such as round or subtle spindle shapes and a low to modest amount of granules — suggested that the mother cells involved in these mitoses are likely of HC, HC/SGC or SGC identity. Divisions of cells with GC characteristics were never observed. Previously published data showed that the immune system releases all labeled cells by days 6 (HPT) and 8 (APC) after a single BrdU injection [5]. Therefore, the rarity of divisions observed in the longitudinal analysis that follows indicates that changes in the percentage of labeled hemocyte types observed after day 8 must be associated with relationships among the HCs, SGCs and GCs – rather than suggesting continuing release of labeled cells from the immune system.

### Longitudinal analyses suggest a lineage relationship among the hemocytes

In order to investigate these developmental relationships among circulating hemocytes, EdU was injected into *P. clarkii* crayfish and hemolymph samples were taken at various time points over the course of the next two months. Hemocyte types were then categorized to track the variations in the frequency of EdU-labeled cells over time. Because there are no reliable hemocyte type-specific visual markers, a combination of qualitative characteristics — namely, presence of granules and morphological features — as well as the previously mentioned quantitative characteristics of cell area and NC ratio, were utilized to categorize the cell types present at each time point. All three hemocyte types (HCs, SGCs and GCs) were found in hemolymph samples in this longitudinal study (Table 1). For the following sections, all EdU-labeled hemocyte type frequencies are reported as the proportion of the total number of cells surveyed per sample, including non-EdU-labeled hemocytes. Data were normalized to accommodate for differences in available hemocyte populations for analysis per animal per time point.

**Table 1:** **Hemocyte count data from the longitudinal study.** Data are separated by cell type and time point. Mean refers to the average normalized frequency of EdU-labeled hemocyte types in the total population of labeled and unlabeled cells in the circulation. A total of 2218 hemocytes are represented in this table

Time Points	Hyaline			Hyaline/Semigranular Transition			Semigranular			Semigraular/Granular Transition			Granular			Total *n* per time point
	Mean (%)	SEM	*n*	Mean (%)	SEM	*n*	Mean (%)	SEM	*n*	Mean (%)	SEM	*n*	Mean (%)	SEM	*n*	
6 h	0.6435	0.2264	41	0.659	0.1905	46	0.1834	0.1207	14	0.0289	0.0191	2	0.0000	0.0000	0	103
3d	2.5430	1.0852	129	1.3469	0.2725	66	0.5667	0.2312	27	0.0600	0.0671	3	0.0000	0.0000	0	225
10d	3.3302	1.0968	89	2.2182	0.6838	62	4.1239	0.5503	118	1.3230	0.1022	37	1.1984	0.4551	34	340
17d	1.7612	0.2741	47	2.9034	0.2254	82	7.4428	1.8745	207	2.5408	0.7000	74	1.6884	0.3125	44	454
31d	0.7064	0.2450	22	1.4309	0.2047	45	3.8349	1.0485	121	1.6870	0.1985	53	4.6174	0.4811	146	387
45d	0.3736	0.1322	10	1.4453	0.3242	39	1.9960	0.1667	51	1.2575	0.1321	33	3.5398	0.4386	89	222
56d	0.1217	0.0253	4	1.3624	0.2597	44	1.5203	0.5100	49	1.1291	0.2892	36	2.3622	0.6540	75	208
63d	0.4136	0.3660	10	1.3273	0.6968	46	1.9152	1.0401	67	1.4473	0.4161	66	1.6666	0.4790	90	279
Total n per cell type			352			430			654			304			478	

**Fig. 4:**
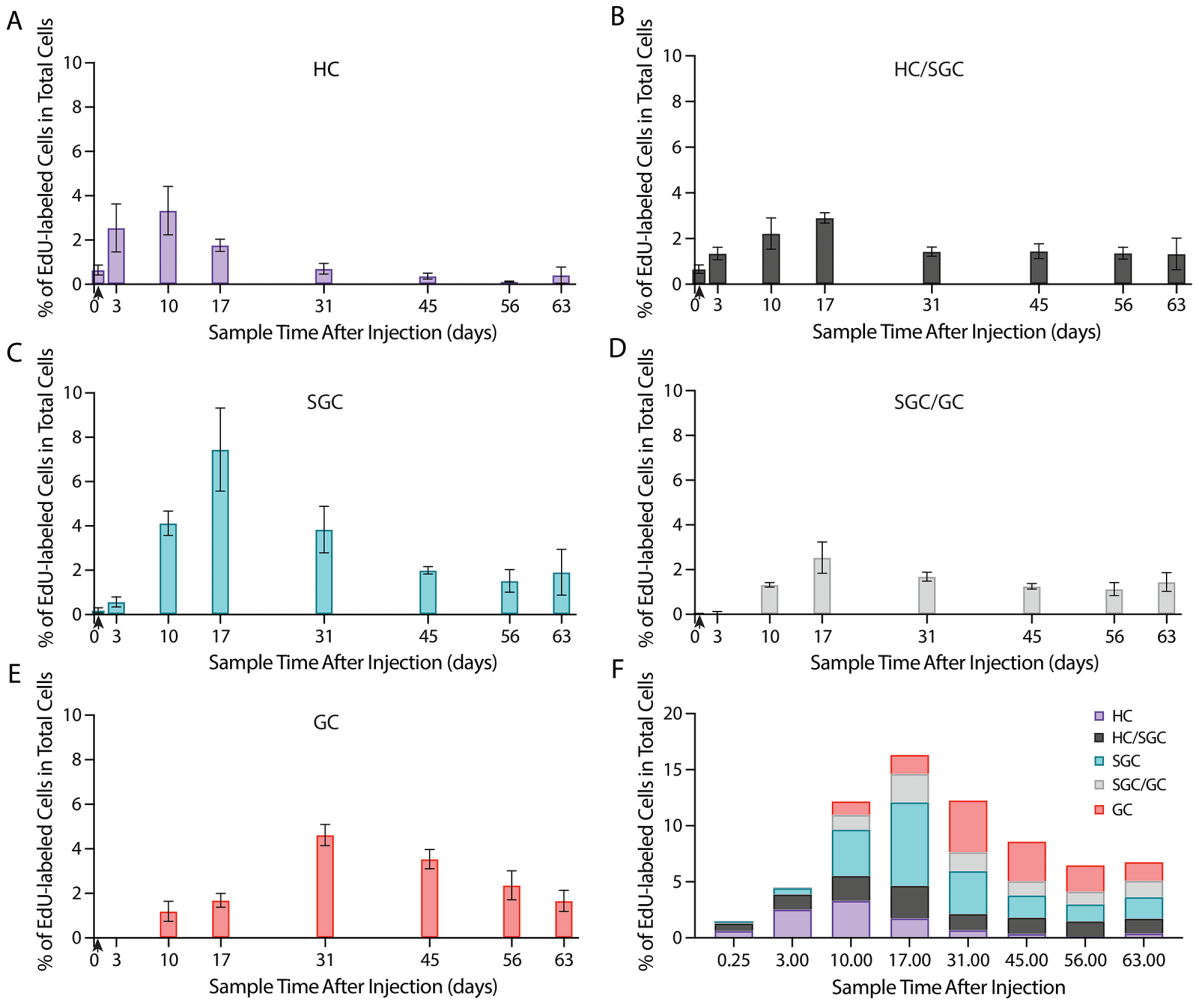
**In vivo longitudinal study of EdU-labeled hemocyte population suggests a single hemocyte lineage that includes all hemocyte types.** Crayfish received a single EdU injection, and hemolymph samples were taken periodically for two months to track the status of EdU-labeled hemocytes. At each time point, each EdU-labeled hemocyte was categorized as one of the five hemocyte types based on morphological characteristics, counts recorded and reported as a percentage of the total number of cells (EdU-labeled and unlabeled) counted. **(A-E)** Each graph shows the proportion of EdU-labeled hemocytes of a single type (indicated on graphs), represented as a percentage of the total number of cells (EdU-labeled and unlabeled) counted. **(F)** Stacked bar graph shows the proportions of each hemocyte type and total percentage of EdU-labeled cells of all cells counted (EdU-labeled and unlabeled). The maximum percentage of EdU-labeled cells occurred at 17 days after EdU injection, and this peak was composed of all three hemocyte types and the two transitional forms. A total of 2218 cells contributed to this data set; normalized frequencies per time point per cell type are provided in Table 1. **(A-E)** Vertical arrowhead on the x axis marks the 6 h time point. Error bars represent S.E.M.

The first hemolymph sample was taken six hours after the injection of EdU. In this sample, the majority of EdU-labeled cells were categorized as HC (0.67% of labeled and unlabeled cells; 39.25% of labeled cells) or HC/SGC (0.69% of labeled and unlabeled cells; 44.86% of labeled cells) (Fig. 4A, B and F). This was followed by an increase in both of these frequencies at 3 days post-injection: HC frequency went up threefold and HC/SGC frequency doubled (Fig. 4A and B), presumably due primarily to the release of labeled cells from the immune system, although cell divisions in the circulation may have also contributed. HCs continued to increase at 10 days post-injection, when they peaked in frequency. This suggests that this time point captured the full release of EdU-labeled cells from immune tissues because there is no residual EdU in the circulation to be taken up by S-phase cells, and all EdU-labeled cells in the source tissue have been released (Fig. 4A). Previous studies have shown that cells labeled with BrdU were fully released from immune tissues by 8 days post-injection [5]. Therefore, at 10 days post-injection, the EdU-labeled population to be followed for the remainder of the study has been established. After the 10d time point, the proportion of EdU-labeled HCs in the hemolymph decreased; at day 31, these HCs comprised less than 1% of all cells and the low frequency was maintained for the remainder of the 63-day study (Fig. 4A and F).

The SGCs were a rarer hemocyte type in the early post-injection hemolymph samples, comprising less than 1% at both the 6 h and 3-day time points but increasing in frequency to 4.12% at 10 days, then to 7.44% at 17 days (Fig. 4C). It is important to note that this peak in SGC frequency at 17 days was about twice the peak in HC frequency at 10 days; at this time point, SGCs comprised 45.59% of all EdU-labeled cells (Fig. 4A and C). Following this 17-day peak, the SGCs began dropping in frequency relatively quickly, until they comprised less than 2% of all cells by 45 days (Fig. 4C). Labeled GCs did not appear in samples until 10 days following the EdU injection, and GCs increased to peak frequency (4.62% of labeled and unlabeled cells; 37.73% of labeled cells) at 31 days (Fig. 4E). At this point, GCs became the most prevalent EdU-labeled hemocyte type and maintained this majority until day 63, when the frequency of GCs dropped enough to become roughly equivalent to SGCs. Unlike the other hemocyte types, GCs did not then rapidly decrease in frequency and hold at sustained lower levels, as the HCs and SGCs did after their major peaks at 10 and 17 days, respectively (Fig. 4A, C and E). Instead, the GCs slowly dropped in frequency until 63 days (Fig. 4E). A Chi-square test of the cell type counts at each time point revealed there is a significant change in the normalized frequency distributions over the longitudinal study (X^2^ (28, *N* = 2218) = 347.11, *p* < 0.0001) (Fig. 4). Further analysis yielded a Cramer’s V of 0.3293, indicating a strong interaction between cell type and time. When transitional states were removed from this analysis, the Cramer’s V increased to 0.5246 but the decreased number of groups compared causes the resulting interaction strength to remain the same.

It is interesting to note that in a separate longitudinal study using the same approach, one crayfish survived for 7 months after injection of the proliferation marker BrdU. In that case, BrdU was still observed in circulating GCs 223 days following injection of the proliferation marker, suggesting that these hemocytes are long-lived. Importantly, these labeled GCs were rare in these hemolymph samples ~7 months after BrdU injection, indicating these are likely the last surviving hemocytes from the original labeled population.

## Discussion

These analyses validate the qualitative and quantitative metrics used to identify different hemocyte types in crayfish. Further, fluctuations in the proportions of different EdU-labeled hemocyte types over time provide insight into relationships among these circulating cells. Finally, these temporal data inform our understanding of the hemocytes that function as primary neural progenitors involved in adult neurogenesis in *P. clarkii*.

### Validation of quantitative metrics for hemocyte identification and classification

The criteria for the classification of hemocytes as hyaline (HC), semigranular (SGC) or granular (GC) established in the early 1990s were largely reliant on qualitative characteristics, such as the presence of pseudopodia or granules, and other characteristics of cell morphology (Fig. 2) [15, 20]. Quantitative metrics, such as NC ratio and whole cell area, have been used on a relative scale, but no specific ranges for different hemocyte types have been reported. The data presented here confirm that NC ratio and whole cell area are significantly different across all hemocyte types in *P. clarkii*, including intermediate states between the HCs, SGCs and GCs (Fig. 3). Due to the substantial overlap between cell types depicted in Fig. 3C, these two measurements ought to be used together to predict a specific cell type in the absence of qualitative data. However, qualitative data are required to confidently classify hemocytes by type. No sexual dimorphisms were observed in these data. Additional research will be required to determine if these specific measurements are broadly applicable to other crayfish species.

A few studies have sought distinguishable molecular characteristics of SGCs and GCs in *C. quadricarinatus* [40] and in *P. leniusculus* [17, 24, 37]. However, the markers discovered in these two species are distinct, and so it appears that there may be significant diversity in the molecular signatures of hemocytes between species. Perhaps for this reason, reagents that can be used to reliably visualize and distinguish hemocyte types in different organisms have not become generally available. Single cell RNA sequencing studies further complicate this story, as these studies suggest far more than the three accepted hemocyte types (or the five — including transitional stages — proposed in this paper). In *P. leniusculus*, Söderhäll et al. (2022) [32] reveal six distinct clusters of hemocytes with loose comparisons to the three established hemocyte types. In *P. clarkii*, Xin and Zhang (2023) [38] propose the existence of 12 hemocyte types, only four of which are involved in immune functions. Two of these four are specifically distinguished as SGCs and GCs, both responsible for producing antimicrobial peptides, and the remaining two are untyped hemocytes involved in cell proliferation and the general immune response [38]. From these studies it is clear that there are many different subtypes, and that hemocytes are even more diverse than had been imagined. Further research is therefore needed to comprehensively understand the molecular identities and mechanisms that differentiate hemocyte types.

### A longitudinal study suggests a hemocyte lineage relationship in *P. clarkii*

A single injection of the proliferation marker EdU was used to label hemocytes in vivo, and subsequent hemolymph samples taken at intervals after injection were used to track the presence of EdU labeling in the entire hemocyte population. This relatively straight-forward approach provided data suggesting direct lineage relationships among the HCs, SGCs and GCs. The earliest time points for hemocyte analysis in this study were 6 h and 3 days post-EdU injection. These are critical timings because the clearing time for the proliferation marker in *P. clarkii* is about 30 h; by then, the levels of freely available EdU are below detection thresholds [6]. Therefore, the 6 h time point provides a glimpse of exactly what types of cells were undergoing S phase at the time of injection. The 3-day time point marks the time at which all of the EdU in the crayfish has been taken up by cells and, as a result, no new labeling can occur and the population of newly labeled cells to be studied longitudinally has been finalized. The one caveat is that release of some labeled cells from the HPT may continue after the EdU clearing time as HPT lineages are completed and cells are released; however, we know that all cells are released by 6 and 8 days from the HPT and APC, respectively. These are critical points to keep in mind regarding the interpretation of the longitudinal data.

EdU-labeled SGCs are also present in the early time points, although labeled HCs are by far the most prevalent cell type at 6 h and 3 days. The presence of labeled SGCs can be explained as circulating cells that were dividing near the end of their HC/SGC stage and matured between the time of injection and hemolymph collection, although release from the HPT as SGCs is also possible until after day 6 when all cells should have been released (Fig. 4C) [5]. At the later time points, it becomes increasingly unlikely that labeled cells would be released from the HPT (Fig. 1B and C in [5]). In conjunction with the presence of the transitional stages in the circulation, these data indicate that the appearance of new EdU-labeled hemocyte types in the samples after day 6 is most likely due to the differentiation of labeled cells already in the circulation.

Most notable about these data is the timing of the maximum frequency peaks of HCs, SGCs and GCs, which occur in that order and in rapid succession (Fig. 4A, C and E). This can be explained by either of two options: (1) hemocytes may compose a single lineage, in which HCs make up the most immature stage, SGCs are a transitory stage and GCs are the most mature and the terminal stage; or (2) hemocytes in the circulation constitute a branched lineage stemming from HCs, in which the branch to SGCs proceeds at an accelerated rate compared to the GC branch. However, when the timing data showing gradual and sequential changes in labeled hemocytes from HCs to SGCs to GCs are combined with the morphological data showing that nucleocytoplasmic ratio and whole cell area are strongly correlated with cell type, our findings more strongly support a single lineage model incorporating all three hemocyte types.

Moreover, the frequencies of the intermediate states — both HC/SGC and SGC/GC — tend to have consistently low values across all time points (less than 3% of all cells counted), suggesting that cells spend relatively little time in these transitional states before maturing into the next cell type (Fig. 4B and D). Due to these stable levels in the intermediate states, the transition rates of HCs to SGCs and SGCs to GCs can be estimated from these data to be about one week and about one to three weeks, respectively.

Finally, the frequency of all EdU-labeled hemocytes in the circulation reaches a peak at 17 days (Fig. 4F). In the 3 day and 10 day samples, the increases in the frequency of EdU-labeled hemocytes are likely due to a combination of continued release of newly generated hemocytes from the HPT and hemocyte divisions in the circulation. However, after the 10-day sample, this trend is likely due solely to cell divisions in the circulation, since release of labeled cells from the immune system is complete. However, day 17 is when the maximum number of EdU-labeled cells are observed, and after that time there are not enough divisions happening in the circulation to continue increasing or to even sustain this value. At this point, the frequency of EdU-labeled HCs is declining and no longer meaningfully contributing daughter cells to the hemocyte population. However, as a result, the only way in which this high total frequency could be maintained would be if SGCs and GCs were also dividing at high rates in the circulation. Because the total frequency of EdU-labeled hemocytes steadily declines between the 17 days and 56 days, SGCs likely divide much less frequently, and SGC/GCs and GCs potentially do not divide in circulation at all, which is consistent with observations of the sampled cells (Fig. 4F).

**Fig. 5:**
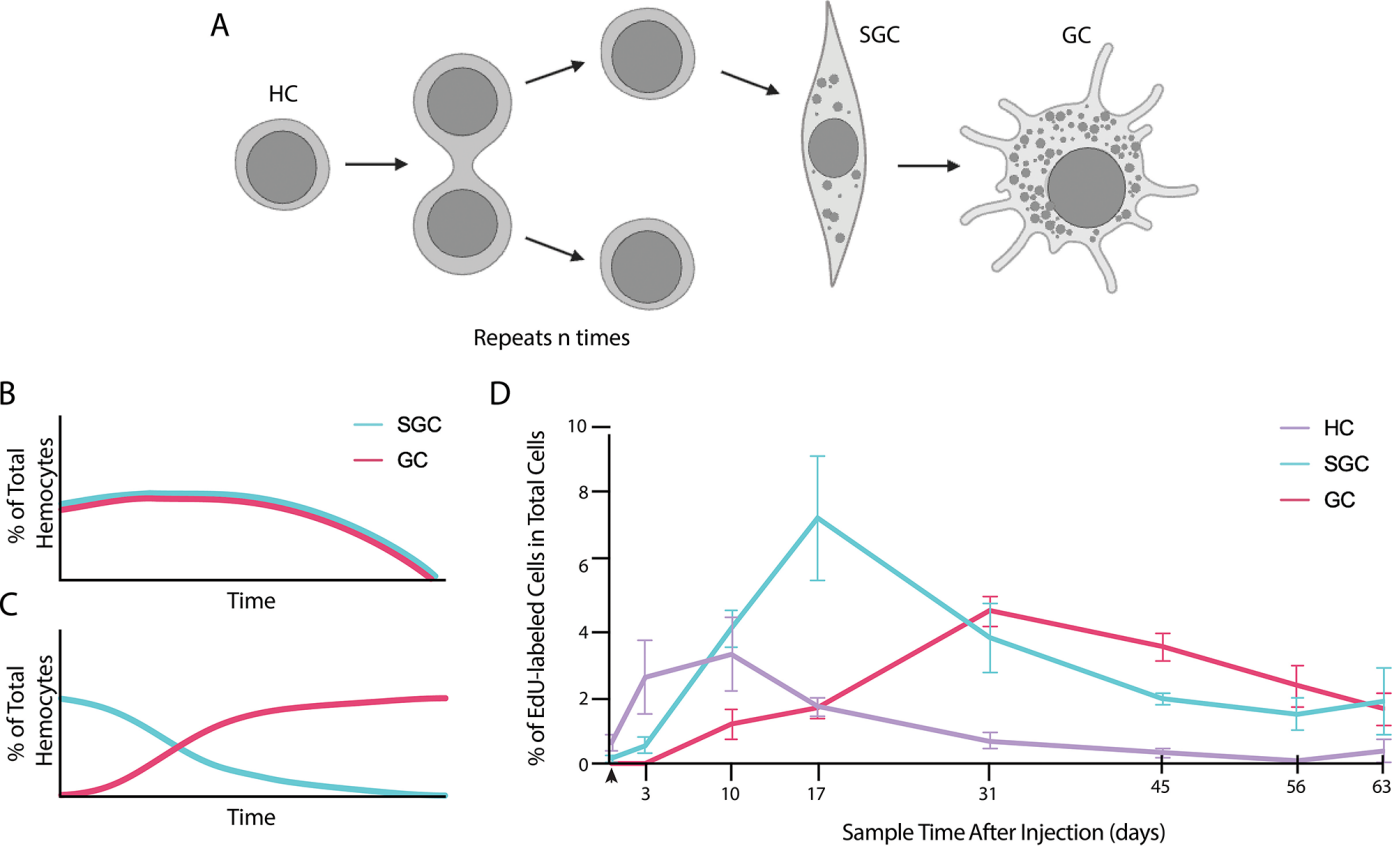
**Proposed hemocyte lineage model in** ***P. clarkii. *(A)** Proposed hemocyte lineage model based on data presented. Hemocytes are released from the HPT as HCs (leftmost cell type). They then divide an unknown number of times in the circulation, producing daughter HCs. HC/SGCs and SGCs (middle cell type) may also have the capacity to divide; however, GCs (far right cell type) have never been observed undergoing mitosis in the circulation. Once the HCs have completed their terminal division, potentially determined by unknown endogenous factors, they begin along a single developmental lineage that includes all three hemocyte types. HCs first mature into SGCs before becoming GCs. Our data indicate that the time spent transitioning from HC to SGC is around one week, while there is likely more variability in the transition time between the SGC and GC hemocyte types, ranging from about one to three weeks. **(B-C)** Predictions of labeled hemocyte type relative frequencies in presumptive longitudinal experiments following a single injection of a proliferation marker, modeled on results reported in **(B)** Lin & Söderhäll, 2011 [23] and **(C)** Li et al., 2021 [22]. HCs are excluded from these models as HCs are not included in either of these data sets. **(D)** Results of current longitudinal study, focusing only on the three main hemocyte types (HCs, SGCs, GCs). Transitional state hemocytes are not included in these numbers. Error bars represent S.E.M.

Our data suggest that HCs initiate the hemocyte lineage leading to SGCs and GCs. We therefore propose the following lineage to accommodate the hemocyte type frequencies observed in this study (Fig. 5A). Hemocytes are released from the HPT as HCs, which will divide a number of times in the circulation to produce daughter HCs. These HCs will eventually mature into SGCs, at which point they may still have the ability to divide, perhaps at a lower frequency. The SGCs continue to mature into GCs, which is their terminal cell fate. No GCs were observed undergoing mitosis. As mentioned earlier, labeled GCs were still observed in a separate longitudinal study 7 months after a single injection of BrdU, although these were very few compared to the numbers of labeled cells we observe in the present study. Nevertheless, this suggests the possibility that GCs can have a long lifespan.

### Hemocyte developmental lineages in *P. leniusculus* and *C. quadricarinat*us

The evidence presented here suggests that the existing lineage models for crayfish hemocytes are not fully representative of what has been observed in *P. clarkii*. Firstly, the presence of an abundance of HCs in the hemolymph is at odds with the lineage models described by both Lin & Söderhäll, 2011 [23] and Li et al., 2021 [22]. The former studied hemocytes in *P. leniusculus* and proposed that SGC and GC hemocytes are released from the HPT with a predetermined cell fate that is reflected in the cellular morphology [23]. In *P. clarkii*, however, the major peak in labeled GCs (day 31 post-injection) does not occur until after release of labeled cells from the immune system has ended by days 6 (HPT) and 8 (APC) [5].

Li et al. (2021) [22] did in vitro experiments with hemocytes from *C. quadricarinatus* suggesting that cells are released from the HPT as immature SGCs, already exhibiting granules and pseudopodia, and that these mature into GCs in the circulation. They do not include HCs in their analysis because they consider them rare in this species [22]. This is in contrast to numerous studies using *P. clarkii* as a model organism that observed the presence of HCs in circulating hemolymph [5, 10, 20, 28].

The average nucleocytoplasmic ratio of the HCs surveyed in this study was 0.624 and the average whole cell area across this population was 69.17 µm^2^, both of which concur with the quantitative metrics of HCs as put forth by Hose et al., 1990 [15] and Lanz et al., 1993 [20]. These results are aligned with extensive literature citing HCs as a critical aspect of the crustacean innate immune system [5, 31]. In addition, HCs are by far the predominant labeled hemocyte type observed at the earliest (6 h post-injection) time point observed in the present studies, indicating that this is likely the primary cell type released from the HPT (and/or APC) in *P. clarkii* [5].

The behavior of the cell populations over time highlights distinctions between our findings and previous lineage data. If the distinct lineages in the HPT proposed by Lin & Söderhäll (2011) [23] reflect the maturation pathways of hemocytes, we would expect in a longitudinal study to see only SGCs and GCs labeled in the hemolymph at the earliest time point (Fig. 5B). Throughout the next several days, due to continued release of these labeled cells from the HPT, the overall frequency of both of these groups would increase, but likely the relative proportions of SGCs and GCs would remain constant assuming these cell types do not divide while in the circulation (Fig. 5B). Then, both of these cell types would gradually die off, as these are each terminal forms for their respective lineages.

If the single lineage proposed by Li et al. (2021) [22] was used to model the outcome of a longitudinal study similar to the one we performed, the first hemolymph sample should reveal an abundance of only immature SGCs (Fig. 5C). Because the maturation of these cells will vary, we would expect to see a combination of mostly mature SGCs with a few GCs within a couple of weeks (Fig. 5C). Over the next two to three months, the frequency of each of these cell types would flip, as the mature SGCs continue to become GCs; finally, only GCs would be present in hemolymph samples at four to five months, according to the in vitro timeline of Li et al. (2021) [22] (Fig. 5C). After these time points, there should no longer be cells labeled with a proliferation marker. However, it is important to stress that this model was generated with in vitro data, and so the progression observed may be altered in its basic features or timing by endogenous factors present in the circulating hemolymph in vivo.

Neither of these proposed lineages reflects the observed fluctuations in the EdU-labeled hemocyte type frequencies in the present study (Fig. 5D). Further, neither of these lineages includes HCs as a possible hemocyte type in the circulation, while HCs are a major factor and the starting point of our model for *P. clarkii*. The previous studies discussed above were performed using different crayfish species and experimental approaches. It is not obvious why our data diverge from the published studies, since these types of mechanisms are often conserved among closely related species. To unify these perspectives, collaboration among labs and similar studies need to be performed in the three species and data compared.

### Which hemocyte is the primary neural progenitor?

The primary neural progenitors in the niche of the adult crayfish brain are capable of both self-renewing and consuming divisions [7]. However, in our experience, the self-renewing divisions are relatively rare, i.e., observed in only 5% of niches in a recent study [7]. We therefore hypothesized that in addition to the self-renewing divisions, the niche progenitor cell pool is also maintained by replenishment from a source extrinsic to the niche. Several approaches have been used to discover the origin of these extrinsic cells. Among these, in vitro studies using desheathed *P. clarkii* brains with exposed niches revealed that hemocytes alone had a remarkable affinity for the niche when compared with other cell types [6]. The location of the niche on top of a blood vessel that has direct access to the niche via a central “vascular cavity,” within a hemolymph-filled space between the brain and sheath, certainly could provide ready access for hemocytes [9]. Further, adoptive transfers of EdU-labeled hemocytes demonstrated that labeled cells appeared in the niche, streams and neuron clusters in the brain, where they expressed appropriate neurotransmitters, suggesting that adoptively transferred EdU-labeled hemocytes can generate cells with neural properties [4]. Perhaps most compelling, adoptive transfers were performed with donor hemocytes that had been EdU labeled and then separated with Percoll gradients into two fractions: layer 1 containing HCs and SGCs, and layer 2 containing GCs [5]. When these hemolymph fractions were independently transferred into naive crayfish, layer 1 hemocytes — but never layer 2 GCs — were later found in the niches of recipients, although the characteristic HC and SGC morphologies were no longer distinguishable. After an interval of several weeks, EdU-labeled cells that also labeled immunocytochemically for appropriate neurotransmitters were found in the brain cell clusters where adult-born neurons are incorporated, as in previous adoptive transfer experiments [4]. These studies demonstrated that either HCs or SGCs could act as neural progenitors. However, taken together with the finding that HCs are the only hemocyte type that incorporates proliferation markers in vitro and in vivo [5], these studies indicated that HCs are the most likely progenitor cell candidates. In contrast, our longitudinal data indicate that EdU labeling, initially found predominantly in the HCs, is later observed in SGCs, making the SGCs potential progenitor candidates since they, too, could have been labeled in the adoptively transferred samples, although at a slower rate than the HCs in the time frame used.

In the present study, the relative frequencies of each hemocyte type following the injection of a proliferation marker can be used in conjunction with previous findings to further examine this question of hemocyte origins of the neural progenitors. In the adoptive transfer experiments described above, hemolymph was drawn for transfer 3–5 days [4] and 7–8 days [5] after EdU injection into donor crayfish. These time frames can be examined in our current data for insight into the prevalence of labeled hemocyte types after injection of EdU. At the 3d time point, HCs and HC/SGC intermediate cells are by far the most frequently labeled cell types: 56.90% and 30.17% of all EdU-labeled hemocytes in circulation, respectively (Fig. 4F). Therefore, any incorporation of labeled cells into the niche with hemocytes from this time point would very likely be either pure HCs or HCs just as they are maturing into an SGC. Over the next week, labeled SGCs increase quickly in proportion to other labeled hemocytes to 34.71%, surpassing that of HCs (26.18%) by the 10 day timepoint (Fig. 4F). However, the proportion of HCs and HC/SGCs combined (44.42%) still makes up the majority of circulating EdU-labeled cells (Fig. 4F). If labeled cells from this time point were to be injected into a recipient animal, it would still be more probable that an HC or HC/SGC hemocyte could be the primary type migrating to the niche. In addition to the presumed incorporation of proliferation markers into developing hemocytes in the HPT, there is also evidence that HCs and HC/SGCs are the primary hemocyte types still active in the cell cycle after release from the HPT, with SGCs potentially also engaging in mitosis though less frequently. Therefore, HCs and HC/SGCs are the most likely cell types to incorporate proliferation markers while in the circulation, explaining why they have higher frequencies of labeling in the earliest time points (Fig. 4).

## Conclusion

These studies were undertaken to examine the hemocyte population in the crayfish *P. clarkii* because our studies indicate that some of these cells act as progenitors in the production of adult-born neurons. We therefore wanted to understand the relationships among the hemocyte types, with the goal of further identifying and characterizing the hemocyte type that is involved in adult neurogenesis. Our specific aims were to: (1) validate quantitative and qualitative metrics as indicators of the traditional categories of hemocytes: hyaline (HCs), semigranular (SGCs) and granular cells (GCs); (2) undertake a longitudinal study of hemocyte types following a single injection of proliferation marker, in order to distinguish between possible developmental relationships among hemocytes; (3) use the longitudinal data to confirm or refute the conclusion from previous studies that HCs are the most likely neural precursor. First, our findings suggest that quantitative metrics are reliable predictors of hemocyte type, and can be combined with qualitative characteristics to accurately identify each of the three major hemocyte types. Second, longitudinal studies of hemocyte labeling following a single injection of proliferation marker indicate that these hemocyte types have a direct lineage relationship, supported by the timing of frequency peaks for each labeled hemocyte type and their juxtaposition to decreased frequencies in earlier-labeled hemocyte types. Both of these observations support the proposal that HCs, which are mitotically active and therefore label with proliferation markers, pass through a transitional state (HC/SGC) to mature into SGCs. Immediately thereafter, the SGCs drop in frequency, coinciding with an increase in SGC/GC and GC frequency, supporting a transition between these cell types, and leading to the terminal GCs that will survive for weeks or months. Finally, the fact that HCs are the predominant labeled cell type in the first ten days after EdU injection, the time when hemolymph samples were taken for adoptive transfers in previous studies, corroborates evidence from previous studies that HCs are the most likely to act as neural progenitors in the production of adult-born neurons.

### Looking ahead

As proposed in the Background, the mitotic HCs examined in the current experiments may mature, leave the cell cycle and perform the immune functions reported in the literature. However, it is also interesting to entertain a revised hypothesis that in addition to immune functions, the HCs are multipotent stem cells involved in the generation of hemocytes and also in the production of adult-born neurons — as implied by these studies. In future studies, it will be important to untangle this story and discover the tissue origin of these fascinating cells.

## Data Availability

The datasets used and/or analyzed during the current study are available from the corresponding author on reasonable request.

## Abbreviations

AC: Anticoagulant
APC: Anterior Proliferation Center
BrdU: 5-Bromo-2’-deoxyuridine
CL: Carapace Length
EdU: 5-Ethynyl-2’-deoxyuridine
GC: Granular Cell
HC: Hyaline Cell
HPT: Hematopoietic Tissue
NC: Nucleocytoplasmic
PBS: Phosphate Buffered Saline
PBTx: Phosphate Buffered Saline with 0.3% Triton X-100
SGC: Semigranular Cell
THC: Total Hemocyte Count
